# Comparative analysis of life cycle assessment of biogas-powered and coal-powered power plant for optimized environmental operation

**DOI:** 10.1016/j.heliyon.2024.e39155

**Published:** 2024-10-10

**Authors:** Raman Kumawat, Lata Gidwani, Kunj Bihari Rana

**Affiliations:** a Department of Renewable Energy, Rajasthan Technical University, Kota, 324010, India; b Department of Electrical Engineering, Rajasthan Technical University, Kota, 324010, India; c Department of Mechanical Engineering, Rajasthan Technical University, Kota, 324010, India

**Keywords:** Biogas, Biogas power, Coal power, Anaerobic digestion, Environmental emissions, Life cycle assessment

## Abstract

The major concerns that mankind faces today are limited reserves of conventional energy, growing energy demand, and environmental pollution. This study depicts a comparative analysis done for the life cycle assessment of the biogas-based plant and coal-based plant designed for Bikaji Foods International Ltd., India. OpenLCA version 1.11.0 software was used with the database ecoinvent 3.3 LCIA methods (ReCiPe Midpoint H) to analyze the environmental impact and investigate the effect of the biogas-based plant and the coal-based plant. The functional unit of 1 MJ of energy generated from biogas and coal was selected to represent the results of the production of 15,271,600 MJ of energy. The results for marine eutrophication, particulate matter formation, photochemical oxidant formation and terrestrial acidification for the biogas-based plant were 734.527 kg N-Eq, 6314.012 kg PM_10_-Eq, 1328.629 kg NMVOC and 3.933E04 kg SO_2_-Eq, respectively. Whereas, for coal-based plant, these values were 4919.442 kg N-Eq, 1.962E04 kg PM_10_-Eq, 6486.987 kg NMVOC and 13.448E04 kg SO_2_-Eq, respectively. The greenhouse gas emissions and fossil depletion from the biogas-based plant were found negligible as compared to the coal-based plant. Overall, it was found that the biogas-based plant has a more remunerative impact on the environment than the coal-based plant. This study recommends that local authorities and industrial communities should invest more and more in increasing the number of biogas plants at domestic as well as commercial levels and secure a clean and green future for coming generations.

## 1 Introduction

The explosive growth of the population has led to a sharp rise in energy demand in recent years. However, conventional energy sources are limited, making it impossible to fully meet the total energy demand [1]. The usage of conventional energy sources is causing significant environmental challenges, such as global warming, environmental pollution, and greenhouse gases [2]. Fortunately, these issues can be addressed by converting to non-conventional energy sources that are abundant in nature and environmentally friendly [3]. Biogas is an especially efficient energy source for producing heat and reducing environmental impact, including greenhouse gas emissions. Notably, biogas ranks as the fourth largest contributor to renewable energy sources for power generation, accounting for approximately 10 % of power generation in India and around 15 % worldwide by 2021 [4]. The end product of biogas production can be used as fertilizer, containing rich nutrients that can improve soil fertility and increase organic matter [5]. The dry bio-slurry can potentially be used as supplemental feed for cattle, pigs and poultry.

The process of anaerobic digestion, which converts cattle dung into biogas, presents a promising way to generate renewable energy while providing numerous environmental benefits [3,6]. By harnessing the power of biogas, energy production can be achieved with fewer emissions and greater environmental performance than traditional coal-fired power generation. Coal remains the primary source of power in India, accounting for 56 % of the country's total power generation by 2014 [7]. Coal-fired power plants provide 70 % of India's energy, ranking the country third in CO_2_ emissions from coal-fired power plants worldwide, after China and the USA by 2015 [8]. In 2021, coal-fired power plants in India alone emitted 1574 MM Tonne (million metric tonnes) of CO_2_ [9], with a 180.57 % increase in CO_2_ emissions from 2000 to 2019, accompanied by a 159.61 % growth in coal consumption for power generation during the same period [9]. Over the past century, the global mean temperature has increased by 0.3–0.6 °C, while sea levels have risen by 10–15 cm. Scientists warn that without effective environmental policies in place to reduce greenhouse gas emissions, global temperatures could rise by 1–3.5 °C, and sea levels could rise by 15–95 cm [[10], [11], [12]]. Therefore, transitioning to more sustainable sources of energy, such as biogas, can help address the urgent need to reduce greenhouse gas emissions and limit the impact of climate change.

The generation of bioenergy through biogas power plants has an impact on the environment, and it is important to comprehensively assess the quantitative and qualitative impacts [13]. Life cycle assessment (LCA) is a valuable tool for environmental management, as it enables a scientific analysis of the environmental impact from the beginning to the end of a product system, and identifies ways to reduce the impact [14,15]. LCA is the most reliable method to forecast the environmental impact of any product system, as it assesses factors such as global warming, ozone layer depletion, toxicity, greenhouse gas emissions, and human health [16,17].

Most of the published research focuses only on the energy analysis of biogas power systems without considering environmental aspects that oppose a complete study [[18], [19], [20]]. As a clean and low-carbon energy source, biogas energy has less environmental impact than the conventional energy source of power generation, such as reducing CO_2_ emissions, preventing global warming, etc. [3,5,21]. Several studies were conducted to assess the environmental impacts of biogas power. One study focuses on the life cycle environmental impact assessment of power generated from biogas produced by the anaerobic digestion of waste and agricultural products [22]. Another study examines the biogas production from waste and its impact on the environment, including greenhouse gas emissions and other environmental impacts [23]. Biogas produced from animal manure produces the greatest reduction in greenhouse gas discharges [24,25].

Previously published studies have compared the environmental impacts of biogas-powered sources to those of natural gas, liquefied petroleum gas, and charcoal [26,27]. There is no comparative study of the life cycle assessment of biogas-powered and coal-powered power plants for environmental aspects available in the reported literature to date. This study aims to compare the environmental emissions from a biogas-powered and a coal-powered power plant (both plants had been used for heating purposes) for an Indian food processing industry (Bikaji Foods International Ltd.) through a life cycle assessment perspective.

## 2 Methodology

**Fig. 1:**
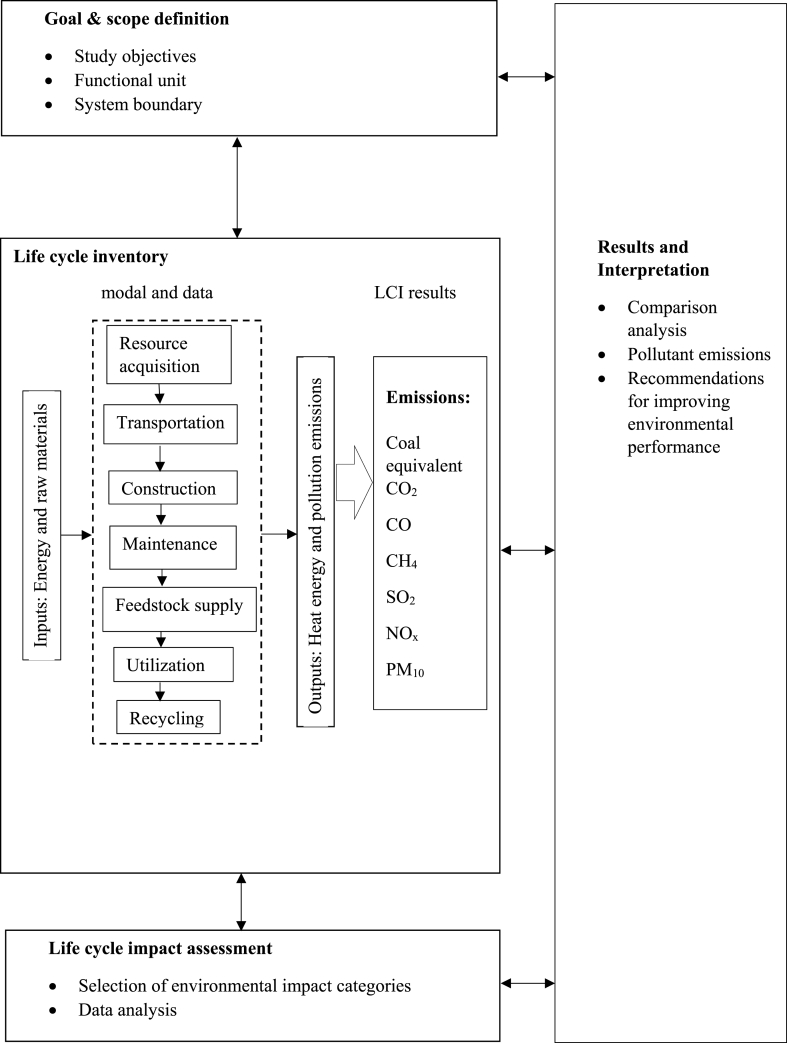
Life cycle assessment framework.

The study evaluates the influence of the proposed biogas plant on various aspects such as fuel consumption, operation, maintenance, feedstock supply, utilization, and digest processing for the food processing industry (Bikaji Foods International Ltd.). The Life Cycle Assessment (LCA) method utilized in this study follows the ISO series (ISO-14040 and ISO-14044) standards [28,29]. The ISO series standards were introduced by the Society of Environmental Toxicology and Chemistry (SETAC). LCA studies are generally divided into two different types; 1) attributional and 2) consequential. The attributional type provides a particular range of functional units while the consequential LCA estimates changes at the functional unit level due to system-wide changes in pollution and resource flows. An attributional LCA describes environmental impacts directly linked to the life cycle of the outcome. In contrast, a consequential LCA describes the magnitude of the assessment, considering minimal information for investigation [30]. In this study, attributional LCA was used because it was assumed that the results of the analysis would not lead to major structural changes in the establishment. This study was done based on statistical analysis. OpenLCA (version 1.11.0) software programme was used for this study, Database used for calculation was ecoinvent 3.3 lica methods 20181205.2 databases and the life cycle impact assessment used the ReCiPe Midpoint H method [31]. Analysis of the results using Monte Carlo simulations showed significant differences in environmental impacts across most categories, pointing to the utility of using the proposed modelling approach for LCI data collection. This method helped in taking some of the following steps in analysing this comparative study: 1) Creation of a Life Cycle Assessment Index System for biogas-powered and coal-powered power plants. 2) Calculation of environmental emissions from biogas-fired and coal-fired power plants. 3) To determine the environmental impact category by interpreting life cycle assessment results of biogas-fired and coal-fired power plants. The International Organization for Standardization (ISO) has developed a comprehensive framework to consistently conduct eco-balance assessments. The key steps of LCA are defining the goal and scope, conducting a life cycle inventory analysis (LCI), carrying out a life cycle impact assessment (LCIA), and interpreting the life cycle results. The life cycle assessment framework is depicted in Fig. 1.

### 2.1 Goal and scope definition

As a goal, this study concentrates on identifying the environmental impacts associated with transitioning from coal-based to biogas-based power generation at Bikaji Foods International Ltd. in Rajasthan state, India. Bikaji Foods International Limited, Bikaner, India was established in the year 1986. At present, the industry's thermal facilities have four coal-based boilers with energy production of 12,552 MJ, 12,552 MJ, 8368 MJ and 8368 MJ (Mega Joule) respectively. Factors such as biogas utilization, feedstock (cow dung), biogas plant construction, and biogas plant maintenance were taken into consideration to determine the environmental impact of biogas production through anaerobic digestion [32]. The scope of the study was defined in line with the regulations of ISO-14040 and ISO-14044. The investigation purpose was carefully defined in detail to ensure the information quality and consistency. The main aspects of this investigation included utilizing cow dung as a feedstock input and biogas production as the sole mode of use. In this study, biogas and a coal-fired power plant were used only for heating purposes.

#### 2.1.1 Functional unit

The functional unit of a product system determines the quantity and performance characteristics of the product, as defined in the ISO-14040 and ISO-14044 standards [28,29]. The functional unit is a key quantitative measure of the performance of the product [32]. In any LCA study, the selection of a representative functional unit is important because it serves as a unifying benchmark to define and quantify the physical and operational characteristics of the product in question [34]. In this study, the functional unit is defined as 1 MJ of energy from biogas and is applied to all aspects of the anaerobic digestion process, including construction, production, feedstock, and utilization. The functional unit is defined as 1 MJ of energy from coal and is applied to all aspects of the mining, including transportation, construction, maintenance, consumption and disposal. The annual heat energy required for all operations at Bikaji Foods International Ltd. is estimated to be 15,271,600 MJ.

#### 2.1.2 System boundary

**Fig. 2:**
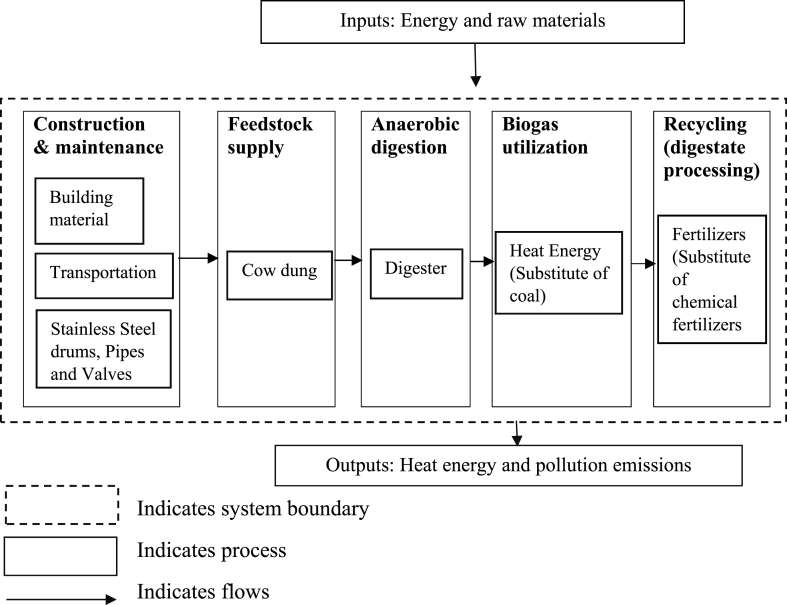
System boundary for biogas-based power generation.

**Fig. 3:**
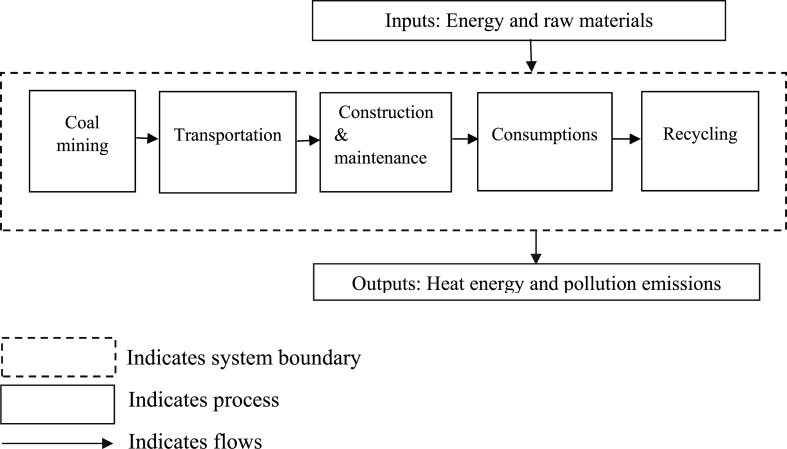
System boundary for coal-based power generation.

In life cycle assessment studies, the system boundary is crucial in determining the input and output activities for a particular system or pathway. This life cycle assessment is based on cradle-to-grave LCA, that is raw material extraction, production, distribution and utilization of biogas and excluding the end-of-life phase. Fig. 2 illustrates the system boundary for biogas-based power generation, which commences from the construction phase of the biogas plant. In this study, cow dung is utilized as feedstock, which is then supplied to the digester to produce biogas as a heat source. Biogas serves as a substitute for traditional energy sources such as coal-based heat. Furthermore, the digestate obtained from biogas production replaces chemical fertilizers since it contains potassium (K), nitrogen (N), and phosphorus (P) values of digestates available from the literature. Fig. 3 shows the system boundary for coal-based power generation, which involves acknowledging the input processes such as raw materials and energy associated with coal acquisition during the manufacturing and production phase. The coal-based power plant generates ash as waste. This ash is used in concrete production, brick and block manufacturing, road construction, agriculture and landfill cover as per the literature [46]. Plant disposal was excluded from the system boundary as the future process of plant disposal remains uncertain.

### 2.2 Life cycle inventory

This phase involves the collection and calculation of data to measure the respective inputs and outputs of the product system [33]. The process of constructing an inventory analysis is iterative. Once data is collected, additional data criteria or deficiencies may be found, and knowledge about the program may be acquired, including modifications to the data accumulation procedures, so that the research goals can be achieved. Data collection and quantification are embedded in the LCI step [33]. The annual energy demand for Bikaji Foods International Ltd. is 15,271,600 MJ. The life cycle inventory analysis of biogas-based energy is presented in Table 1. Currently, the plant operates on coal-based (Indonesian coal) energy, and an estimated 700 tonnes of coal are required annually to meet the energy demand [35]. The life cycle inventory analysis of coal-based energy is presented in Table 2.

**Table 1:** Annual life cycle inventory analysis of biogas-based power [38].

Emissions (kg)	Biogas plant construction	Biogas plant maintenance	Feedstock supply	Biogas utilization	Digested processing	Whole system
**CO**_**2**_	−106,985.194	−72,089.588	1,568,759.838	1,285,604.521	1,335,819.069	4,011,093.376
**CH**_**4**_	−12.217	−1.527	16,861.374	1466.074	5108.350	23,423.580
**CO**	−192.422	−58.032	94,444.156	1524.106	1487.454	97,205.261
**SO**_**2**_	−255.036	−19.853	421.496	5797.099	10,442.720	16,386.427
**NO**_**x**_	−207.694	−99.265	2603.808	9185.867	5780.300	17,266.071
**NH**_**3**_	0.000	0.000	7983.992	0.000	1505.780	9491.299
**TN**	0.000	0.000	1293.505	0.000	0.000	1293.505
**TP**	0.000	0.000	1096.500	0.000	0.000	1096.500
**VOC**	−4.582	−1.527	16,117.647	32.070	439.822	16,519.290
**PM**_**10**_	−364.991	−133.336	5923.854	861.655	4156.930	18,197.639

Here, 0.000 indicates negligible emission; Negative value indicates emission release or energy consumption; Positive value indicates emissions mitigation or energy production; TN: Total nitrogen; TP: Total phosphorus; VOC: Volatile organic compound; PM_10_: Particles on the order of ∼10 μm.

**Table 2:** Annual life cycle inventory analysis of coal-based power.

Emissions (kg)	Coal Mining [41]	Construction [42]	Transport [43]	Coal Consumption [43]	Solid waste Disposal [42]	Whole system
**CO**_**2**_	29,432,272.727	1004.999	362,104.908	2,256,421.321	3605.794	32,055,409.750
**CO**	49,055.999	0.248	89.044	199.379	0.891	49,345.561
**SO**_**2**_	20,877.999	2.864	1018.107	3181.583	10.182	25,090.735
**NO**_**x**_	–	1.425	509.053	1696.844	5.091	2212.413
**N**_**2**_**O**	8122.909	4.649	1696.844	1908.950	16.968	11,750.320
**NH**_**3**_	44,145.090	–	–	–	–	44,145.090
**CH**_**4**_	12,263,999.999	4.655	1696.844	42.421	16.969	12,265,760.890
**H**_**2**_**O**	14,719.455	–	–	–	–	14,719.455
**PM**_**10**_	11,089,363.636	100.897	37,542.683	509.053	336.943	11,127,853.210

This study mainly discusses six types of emissions generated by biogas-based and coal-based power systems during their life cycle. It includes CO_2_ (carbon dioxide), CO (carbon monoxide), CH_4_ (methane), SO_2_ (sulphur dioxide), NO_x_ (nitrogen oxide), and PM_10_ (particulate matter) emissions. The quantification of these emissions is shown in Table 1, Table 2. As evident from Table 1, CO_2_ emissions are the largest emissions from biogas-based power systems and stand at approximately 4,011,093.376 kg. From a life cycle perspective, there were mainly four types of emissions generated in the feedstock supply phase of the biogas-based power system, accounting for 39.11 % of the total emissions of CO_2_, 71.98 % of the total emissions of CH_4_, 97.16 % of the total emissions of CO and 32.55 % of the total emissions of PM_10_. In contrast, SO_2_ emitted in the digested slurry processing phase accounted for 63.73 % of the total emissions, and NO_x_ emitted in the biogas utilization phase accounted for 53.20 % of the total emissions. As evident from Table 2, CO_2_, CO, SO_2_, CH_4,_ and PM_10_ are mainly produced in the coal mining phase of the coal-based power system, accounting for 91.82 %, 99.41 %, 83.21 %, 99.99 % and 99.65 % of its total emissions, respectively. NO_x_ emitted at the coal consumption phase accounted for 14.44 % of the total emissions.

#### 2.2.1 LCI of biogas plant construction

This stage involves the procurement of construction materials and their transportation. The energy demand of the industry under study is 15,271,600 MJ per year, which can be met by 1,106,680 m^3^ of biogas (assuming a burner efficiency of 60 % and a heating value of 23 MJ-m^−3^). The quarry, which is made of stone and brick masonry, has a volume of 5056 m^3^, while the biogas holder, made of steel, has a volume of 1820 m^3^ [35]. The biogas plant utilizes both PVC (Polyvinyl chloride) and cemented pipes. All the necessary construction materials are transported to the construction site via trucks. The diesel consumption rate by these trucks is 0.40 L kg^−1^. The air emissions resulting from the extraction and transportation of building materials were determined through a review of relevant studies [[36], [37], [38]]. The assumed lifespan of a biogas plant is 25 years, and the annual feedstock supply to the digester is 33,215 m^3^ [35].

#### 2.2.2 LCI of biogas plant maintenance

It needs to be maintained to eliminate all the problems affecting the biogas plant during operation. Regular maintenance is essential to address any issues that may arise during biogas plant operation, such as gas and water leakage and gasholder drum rusting. Maintenance is required once a year and includes materials such as cement, gravel, paint, and grease. Transport emissions are calculated similarly to the construction phase.

#### 2.2.3 LCI of feedstock supply

The biogas plant employs cow dung as its feedstock and requires 18,084.290 tonnes year^−1^ of cow dung to meet its energy demand [35]. The plant generates emissions of CO_2_, CO, SO_2_, and NH_3_(Ammonia), totalling 1,568,759.838 kg, 94,444.156 kg, 421.496 kg, and 7983.992 kg, respectively, as shown in Table 1. It also produces 1096.501 kg of total phosphorus, 1293.505 kg of total nitrogen, and 16,117.647 kg of volatile organic compounds as shown in Table 1. The cow dung is available 20 km (kilometre) away from the plant, and transportation of the feedstock consumes a significant amount of energy [35]. The cow dung is procured from a distance of about 20 km so the emissions from transportation are also included in this. This was the reason that the emissions of the feedstock supply phase were higher than those of the other phases. The transport was calculated according to the methodologies applied in the preceding two phases.

#### 2.2.4 LCI of biogas utilization

In addition to being an energy source, biogas is a valuable substitute for coal. The annual production of biogas is 1,106,680 m^3^, as the biogas plant operates continuously for about 300–330 days per year [35]. The environmental impact of the combustion of biogas during the heating process was considered. The analysis of the environmental benefits of biogas usage is based on energy production and the reduction of emissions resulting from the replacement of traditional heat sources such as coal by the industry. During the biogas utilization phase, emissions of CH_4_, CO_2_, SO_2_, CO, and NO_x_ into the air are 1466.075 kg, 1,285,604.521 kg, 5797.099 kg, 1524.106 kg, and 9185.867 kg, respectively, as shown in Table 1.

#### 2.2.5 LCI of digestate processing

The digester produces 5425.360 tonnes of slurry annually, which is nutrient-rich and can replace chemical fertilizers [35]. The high concentration of nitrogen, potassium, and phosphorous in the digested slurry makes it an advantageous fertilizer for the agriculture sector [39]. The average nutrient content in the digested solution includes 1.5–2.0 % nitrogen, 1 % potassium, and 1.0 % phosphorus [40]. The emissions generated from the digestion processing phase include CO_2_, CO, CH_4_, NH_3_, and PM_10_, with quantities of 13,35819.069 kg, 1487.453 kg, 5108.350 kg, 1505.780 kg, and 4156.930 kg, respectively, as shown in Table 1.

### 2.3 Life cycle impact analysis

For this study, the ReCiPe Midpoint (H) impact assessment method was selected to calculate the environmental impact. This is a globally accepted and reliable method for life cycle assessment due to its precision, wide range of impact options, and effectiveness [44,45]. It includes 18 midpoint impact categories, including freshwater ecotoxicity-FETPinf, human toxicity-HTPinf, marine ecotoxicity-METPinf, marine eutrophication-MEP, particulate matter formation-PMFP, photochemical oxidant formation-POFP, and terrestrial acidification-TAP100 for biogas-based power systems. A similar type of impact category was assessed for both coal-based power systems and biogas-based power systems.

## 3 Results and discussion

The impact counts for all chosen impact categories are displayed in Table 3, Table 4. The evaluation of impact at individual stages was assessed. Positive and negative impact category results were obtained; a positive value indicates a decreased effect on the environment, while a negative value indicates a reduced environmental impact.

**Table 3:** Characteristic results for energy produced (15,271,600 MJ) through a biogas-based power system.

Impact Category	Unit	Biogas plant construction	Biogas plant maintenance	Feedstock supply	Biogas utilization	Digested processing	Whole System
**Agricultural land occupation - ALOP**	m^2^a	0.000	0.000	0.000	0.000	0.000	0.000
**Climate change - GWP100**	kg CO_2_-Eq	0.000	0.000	0.000	0.000	0.000	0.000
**Fossil depletion – FDP**	kg oil-Eq	0.000	0.000	0.000	0.000	0.000	0.000
**Freshwater ecotoxicity - FETPinf**	kg 1,4-DCB-Eq	0.000	0.000	168.610	0.000	0.000	168.610
**Freshwater eutrophication - FEP**	kg P-Eq	0.000	0.000	0.000	0.000	0.000	0.000
**Human toxicity – HTPinf**	kg 1,4-DCB-Eq	0.000	0.000	1.625E7	0.000	0.000	1.625E7
**Ionising radiation - IRP_HE**	kg U235-Eq	0.000	0.000	0.000	0.000	0.000	0.000
**Marine ecotoxicity - METPinf**	kg 1,4-DCB-Eq	0.000	0.000	1.117E4	0.000	0.000	1.117E4
**Marine eutrophication - MEP**	kg N-Eq	0.000	0.000	734.527	0.000	0.000	734.527
**Metal depletion - MDP**	kg Fe-Eq	0.000	0.000	0.000	0.000	0.000	0.000
**Natural land transformation - NLTP**	m^2^	0.000	0.000	0.000	0.000	0.000	0.000
**Ozone depletion – ODPinf**	kg CFC-11-Eq	0.000	0.000	0.000	0.000	0.000	0.000
**Particulate matter formation - PMFP**	kg PM_10_-Eq	−51.007	−3.970	2639.176	1159.419	2570.393	6314.012
**Photochemical oxidant formation - POFP**	kg NMVOC	−20.678	−1.609	34.175	470.035	846.707	1328.629
**Terrestrial** a**cidification - TAP100**	kg SO_2_-Eq	−255.035	−19.853	1.998E4	5797.099	1.413E4	3.933E4
**Terrestrial ecotoxicity - TETPinf**	kg 1,4-DCB-Eq	0.000	0.000	0.000	0.000	0.000	0.000
**Urban land occupation - ULOP**	m^2^a	0.000	0.000	0.000	0.000	0.000	0.000
**water depletion - WDP**	m^3^	0.000	0.000	0.000	0.000	0.000	0.000

**Table 4:** Characteristic result for energy produced (15,271,600 MJ) through a coal-based power system.

Impact Category	Unit	Coal Mining	Coal transportation	Coal consumption	Construction	Solid waste	Whole system
**Agricultural land occupation – ALOP**	m^2^a	0.000	0.000	0.000	0.000	0.000	0.000
**Climate change - GWP100**	kg CO_2_-Eq	3.185E7	8.677E5	5.688E5	1385.375	5056.583	3.329E7
**Fossil depletion – FDP**	kg oil-Eq	3.188E5	0.000	0.000	0.000	3.188E5	6.376E5
**Freshwater ecotoxicity - FETPinf**	kg 1,4-DCB-Eq	0.000	0.000	0.000	0.000	0.000	0.000
**Freshwater eutrophication - FEP**	kg P-Eq	0.000	0.000	0.000	0.000	0.000	0.000
**Human toxicity – HTPinf**	kg 1,4-DCB-Eq	0.000	0.000	0.000	0.000	0.000	0.000
**Ionising radiation - IRP_HE**	kg U235-Eq	0.000	0.000	0.000	0.000	0.000	0.000
**Marine ecotoxicity – METPinf**	kg 1,4-DCB-Eq	0.000	0.000	0.000	0.000	0.000	0.000
**Marine eutrophication - MEP**	kg N-Eq	4061.348	198.021	660.072	0.000	0.000	4919.442
**Metal depletion - MDP**	kg Fe-Eq	0.000	0.000	0.000	0.000	0.000	0.000
**Natural land transformation - NLTP**	m^2^	0.000	0.000	0.000	0.000	0.000	0.000
**Ozone depletion – ODPinf**	kg CFC-11-Eq	0.000	0.000	0.000	0.000	0.000	0.000
**Particulate matter formation - PMFP**	kg PM_10_-Eq	1.830E4	315.613	1009.622	0.572	2.036	19,629.844
**Photochemical oxidant formation - POFP**	kg NMVOC	3930.162	591.602	1954.810	0.232	0.082	6476.889
**Terrestrial** a**cidification - TAP100**	kg SO_2_-Eq	1.290E5	1303.176	4131.815	2.863	10.181	134,481.037
**Terrestrial ecotoxicity - TETPinf**	kg 1,4-DCB-Eq	0.000	0.000	0.000	0.000	0.000	0.000
**Urban land occupation – ULOP**	m^2^a	0.000	0.000	0.000	0.000	0.000	0.000
**water depletion - WDP**	m^3^	0.000	0.000	0.000	0.000	0.000	0.000

### 3.1 Freshwater ecotoxicity-FETPinf

Freshwater is a crucial element in the global ecosystem, with disruptions in the concentration of freshwater habitats, water pollution, and composition affecting it. Table 3 shows that the whole biogas-based power system's freshwater ecotoxicity was 168.610 kg 1,4-DCB-Eq. This ecotoxicity value includes emissions of pollutants such as CH_4_, SO_2_, NO_x_, etc., which can contaminate the soil and air.

### 3.2 Human toxicity-HTPinf

The substantial amounts of chemicals and air pollution are causing harmful effects on human health, resulting in the human toxicity impact category. The whole biogas-based power system had a human toxicity impact of 1.625E07 kg 1,4-DCB-Eq, as shown in Table 3. This impact occurs during the feedstock supply stages of the biogas-based power system due to the combustion of diesel during the transportation of cow dung to the biogas plant. Additionally, the production of materials used in the construction of biogas plants releases organic toxic chemicals such as volatile organic compounds, ethane, arsenic ions, polycyclic aromatic compounds, and benzene into the environment through wastewater discharge.

### 3.3 Marine ecotoxicity-METPinf

The impact of a harmful compound on the marine environment is determined by chemical and atmospheric contaminants. The total marine ecotoxicity of the biogas-powered system represented as 1.117E04 kg 1,4-DCB-Eq, is provided in Table 3. The provisioning of raw materials is a primary source of marine ecotoxicity. The manufacture of the (non-ferrous) materials used in the construction of the biogas facility generates numerous harmful substances, in addition to untreated effluent.

### 3.4 Marine eutrophication-MEP

**Fig. 4:**
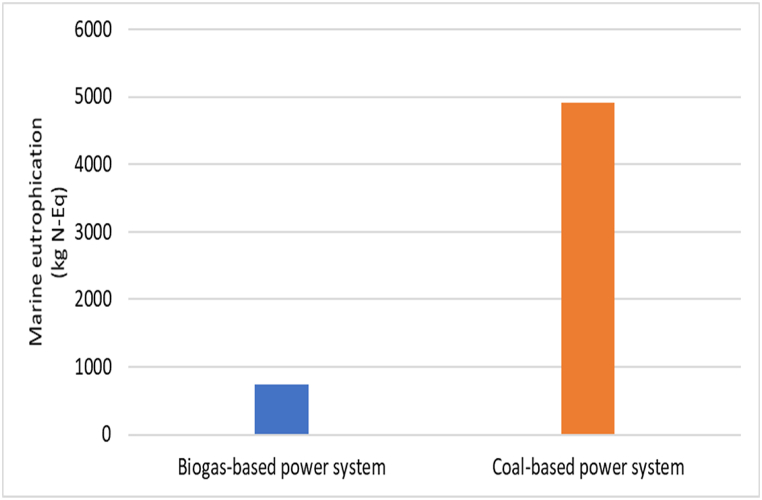
Marine eutrophication-MEP impact for biogas and coal-based power system.

The biogas-based power system results in marine eutrophication of 734.527 kg N-Eq as shown in Table 3. Inorganic nutrients such as nitrogen and phosphorus are major factors responsible for marine eutrophication. The use of organic fertilizers as a replacement for chemical fertilizers in agriculture can help minimize nitrogen emissions in the soil. However, during the feedstock supply stage of the biogas-powered system, nitrogen was discharged into the atmosphere, resulting in environmental deterioration. As depicted in Fig. 4, marine eutrophication is significantly more prevalent in coal-powered systems than in biogas-based power systems. Table 4 reveals that the entire coal-based power system was marine eutrophication of 4919.442 kg N-Eq.

### 3.5 Particulate matter formation-PMFP

**Fig. 5:**
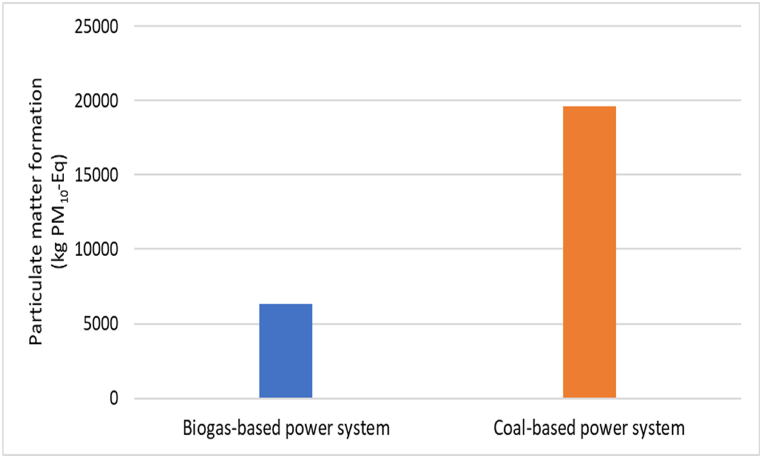
Particulate matter formation-PMFP impact for biogas and coal-based power system.

The particulate matter formation in the biogas-based power system is 6314.012 kg PM_10_-Eq as shown in Table 3. This formation is triggered by the release of SO_2_, NH_3_, and NO_x_ into the air. The production of construction materials such as concrete, cement, steel, and bricks for the biogas plant and their transportation also contribute to the formation of particulate matter, which is discharged into the environment and severely affects human respiratory health. Table 4 shows that the particulate matter formation for the entire coal-based power system is 1.962E04 kg PM_10_-Eq, with coal mining being the primary source at 1.830E04 kg PM_10_-Eq. As illustrated in Fig. 5, particulate matter formation is significantly greater in a coal-based power system than in a biogas-based power system. This highlights the importance of transitioning towards cleaner sources of energy to reduce the emission of harmful particulate matter into the environment.

### 3.6 Photochemical oxidant formation-POFP

**Fig. 6:**
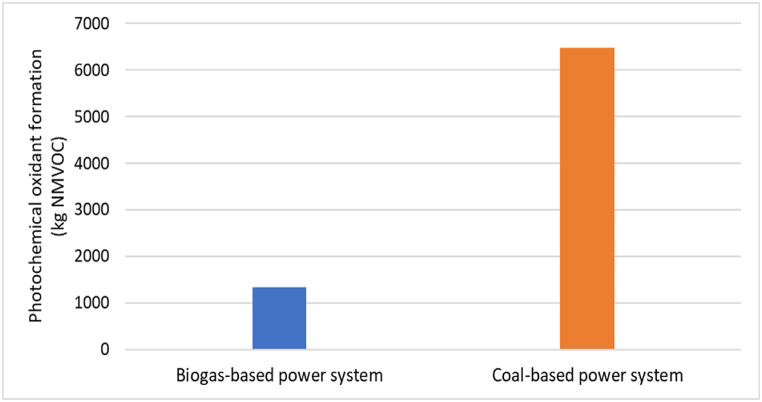
Photochemical oxidant formation-POFP impact for biogas and coal-based power system.

The biogas-based power system generates photochemical oxidant formation of 1328.629 kg NMVOC as shown in Table 3. Photochemical oxidants like aldehydes and peroxyacyl nitrates are formed when nitrogen oxides and volatile organic compounds react with sunlight, and they can significantly damage crops and human respiratory health. As shown in Fig. 6, the formation of photochemical oxidants is substantially higher in a coal-based power system compared to a biogas-based power system. Table 4 displays that the photochemical oxidant formation for the entire coal-based power system is 6486.987 kg NMVOC. This data indicates the need to transition from coal-based power to cleaner sources to mitigate the harmful effects of photochemical oxidants on human health and the environment.

### 3.7 Terrestrial acidification - TAP100

**Fig. 7:**
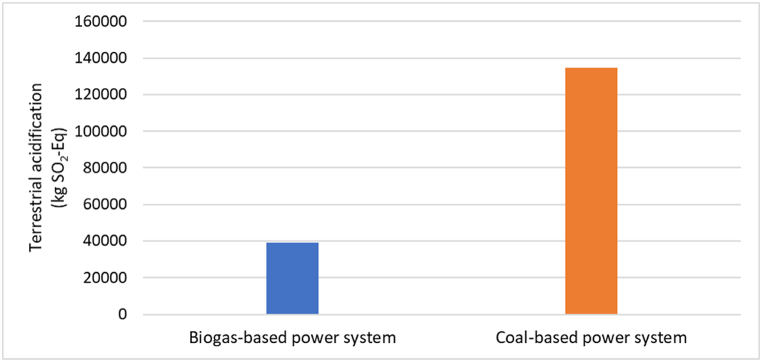
Terrestrial acidification-TAP100 impact for biogas and coal-based power system.

The entire biogas-based power system contributes 3.933E04 kg SO_2_-Eq to terrestrial acidification as shown in Table 3. Terrestrial acidification is a consequence of the release of NH_3_, NO_2_, and SO_2_ into the environment, as well as the use of chemical fertilizers. Construction and maintenance of biogas plants were a negative terrestrial acidification value since they avoid conventional energy sources and chemicals. However, the supply of feedstock and processing of digestate are major sources of terrestrial acidification. In comparison, Table 4 shows that the coal-based power system generates terrestrial acidification of 13.448E04 kg SO_2_-Eq, which is significantly higher than the biogas-based power system. This information is represented in Fig. 7, emphasizing the need for transitioning towards more sustainable energy systems that minimize terrestrial acidification.

### 3.8 Climate change-GWP100

The biogas-based power system had a negligible impact on the environment with zero greenhouse gas emissions, as demonstrated in Table 3. However, the coal-based power system is responsible for emitting 3.329E07 kg CO_2_-Eq of greenhouse gases, as indicated in Table 4.

### 3.9 Fossil depletion-FDP

Zero fossil depletion for the biogas-based power system due to the avoidance of chemical fertilizers and conventional energy sources as shown in Table 3. However, Table 4 shows fossil depletion of 6.373E05 kg oil-Eq for the coal-based power system.

### 3.10 Whole life cycle assessment

**Fig. 8:**
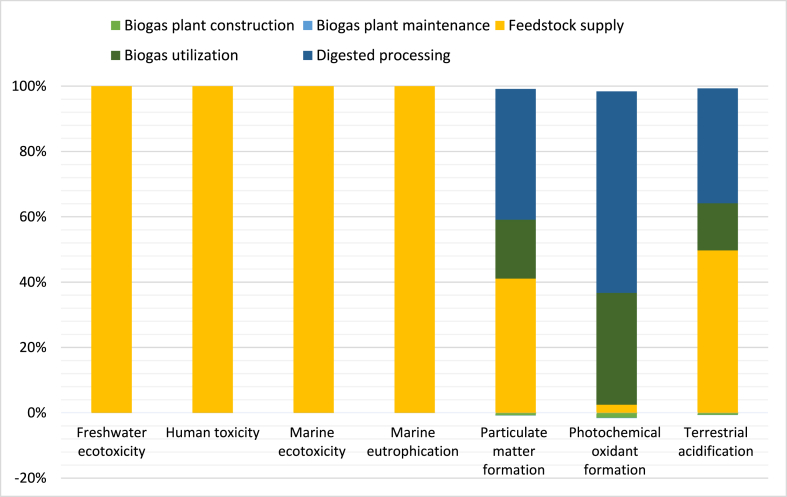
Whole life cycle impact assessment for the biogas-based power system.

**Fig. 9:**
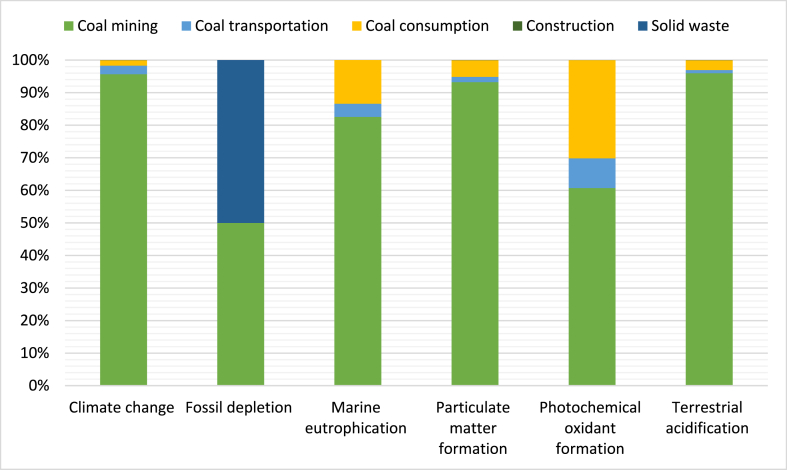
Whole life cycle assessment for the coal-based power system.

The complete evaluation of the biogas-based power system features is presented in Table 3. All outcomes are calculated based on the yearly heat energy production of 15,271,600 MJ to meet the absolute demand. This study assumes a plant lifespan of 25 years and disregards plant site preparation. The findings demonstrate the superiority of the biogas-based system over coal-based alternatives. Notably, the particulate matter formation-PMFP, photochemical oxidant formation-POFP, and terrestrial acidification-TAP100 exhibit negative values during the construction and maintenance phases of the biogas-based power system. The entire life cycle assessment of the biogas-based power system is illustrated in Fig. 8. Over the entire plant life, impacts associated with freshwater ecotoxicity, human toxicity, marine ecotoxicity and marine eutrophication at the feedstock supply phase were high due to the combustion of diesel used to transport cow dung in a biogas power plant and the remaining phases represent zero. The particulate matter formation impact was negative in the biogas plant construction phase and positive in the biogas plant maintenance, biogas utilization and digester processing phases. The production and transportation of construction materials for biogas plants triggers the formation of particulate matter. The photochemical oxidant formation impact was negative in the biogas plant construction phase and positive in the biogas plant maintenance, biogas utilization and digester processing phases. Photochemical oxidants form when NO_x_ and volatile organic compounds react with sunlight. Terrestrial acidification impacts were higher at the feedstock supply and digestate processing phases. Which was due to the release of particulate matter, NOx and SOx into the atmosphere. The terrestrial acidification impact in the construction and maintenance phase of the biogas plant were negative, mainly due to the avoidance of electricity and artificial fertilizers. The entire life cycle assessment of the coal-based power system is demonstrated in Fig. 9. Over the entire life of the coal-based power plant, the impact was high at the coal mining, coal transportation and coal consumption phases due to emissions of pollutants like CH_4_, SO_2_, NO_x_, etc., which can highly contaminate the air, water and soil. While the impact on coal-based power plant construction and solid waste phase was less.

## 4 Conclusion

Life cycle assessment helps identify the key components that impact the environment. The emission of harmful gases from all phases of coal-based power plants is significantly higher than from all phases of biogas-based power systems. For instance, the annual CO_2_ emission from coal-based power systems is 32,055,409.750 kg, while biogas-based power systems emit 4,011,093.376 kg.

The construction and maintenance phases of the biogas-based power system emit fewer harmful gases, indicating a positive impact on the environment. However, emissions from the feedstock supply phase are the largest contributor to the whole life cycle assessment of the biogas-based power system. On the other hand, emissions from coal mining, transportation, and consumption phases are major contributors to the whole life cycle assessment of coal-fired power systems.

The coal-based power system emits significantly higher amounts of harmful gases contributing to climate change-GWP100, fossil depletion-FDP, marine ecotoxicity-METPinf, marine eutrophication-MEP, particulate matter formation-PMFP, photochemical oxidant formation-POFP, and terrestrial acidification-TAP100, as compared to the biogas-based power system. The terrestrial acidification and marine eutrophication from the biogas-based power system are higher than the coal-based system, with 734.527 kg N-Eq and 3.933E4 kg SO_2_-Eq respectively.

Overall, the biogas-based power system is a more environmentally viable option compared to the coal-based system. All phases of the biogas-based power system, including construction, maintenance, feedstock supply, biogas utilization, and digester processing, contribute equally to energy generation. The biogas-based power system's consequences are beneficial to the environment compared to the coal-based system. The authors hope that the issues identified and discussed in this paper can provide insight for other researchers and help set research priorities to support this important research and policy area. This study recommends that local authorities and industrial communities should invest more and more in increasing the number of biogas plants at domestic as well as commercial levels and secure a clean and green future for coming generations. The present study is carried out with five stages of a biogas-based power system such as operation and maintenance, feedstock supply, anaerobic digestion, biogas utilization and recycling at the system boundary, storage and scrubbing at the system boundary, which may be the scope of the further research. The following are some recommendations and ways to proceed.

• In the Indian scenario, most of the industries are associated with coal-based power systems, hence coal is imported to meet the demand. This shift to the biogas-based power system will ensure economic benefits and environmental protection.
• To achieve a reduction in CO_2_ emissions and a green transition in the energy sector, governments should increase the delivery of biogas-based energy by implementing policy measures, such as priority planning for the development of bioenergy systems.
• Supportive policies like subsidies or tax cuts and support for technological innovations are suggested to accelerate the generation and adaptation of biogas in India.

## CRediT authorship contribution statement

**Raman Kumawat:** Writing – original draft, Visualization, Software, Methodology, Data curation, Conceptualization. **Lata Gidwani:** Writing – review & editing, Visualization, Supervision, Project administration. **Kunj Bihari Rana:** Writing – review & editing, Visualization, Validation, Supervision, Resources, Project administration.

## Data availability

The data used in this research are cited in the manuscript.

## Declaration of competing interest

The authors declare the following financial interests/personal relationships which may be considered as potential competing interests:Raman Kumawat reports administrative support and writing assistance were provided by Rajasthan Technical University. Kunj Bihari Rana reports a relationship with Rajasthan Technical University that includes: employment. Raman Kumawat has patent pending to no agency. No other information.

## References

[1] Yolcan O.O. (2023). World energy outlook and state of renewable energy: 10-Year evaluation. Innovation and Green Development.

[2] Koyani K., Shah M., Parikh S.P., Shah D. (2023). A systematic study on simulation and modeling of a solar biogas reactor. Environ. Sci. Pollut. Control Ser..

[3] Shaibur M.R., Husain H., Arpon S.H. (2021). Utilization of cow dung residues of biogas plant for sustainable development of a rural community. Current Research in Environment Sustainability.

[4] Kaur G., Sharma N.K., Kaur J., Bajaj M., Zawbaa H.M., Turky R.A., Kamel S. (2021). Prospects of biogas and evaluation of unseen livestock based resource potential as distributed generation in India. Ain Shams Eng. J..

[5] Zhang C., Xu Y. (2020). Economic analysis of large-scale farm biogas power generation system considering environmental benefits based on LCA: a case study in China. J. Clean. Prod..

[6] Rehl T., Müller J. (2011). Life cycle assessment of biogas digestate processing technologies. Resour. Conserv. Recycl..

[7] Agarwal K.K., Jain S., Jain A.K., Dahiya S. (2014). A life cycle environmental impact assessment of natural gas combined cycle thermal power plant in Andhra Pradesh, India. Environmental Development.

[8] Mishra M.K., Khare N., Agrawal A.B. (2015). Scenario analysis of the CO_2_ emissions reduction potential through clean coal technology in India's power sector: 2014–2050. Energy Strategy Rev..

[9] Independent Statistics & Analysis U.S. Energy Information Administration (EIA). https://www.eia.gov/international/data/world/otherstatistics/emissionsbyfuel?pd=40&p=00000000000000000000000000000000000000000000000000000008&u=0&f=A&v=mapbubble&a=&i=none&vo=value&t=C&g=00000000000000000000000000000000000000000000000001&l=249ruvvvvvfvtvnvv1vrvvvvfvvvvvvfvvvou20evvvvvvvvvvnvvvs0008&s=662688000000&e=1514764800000&.

[10] Dahlman L., Lindsey R. (2024). Climate change: global temperature, NOAA Climate.gov, Science and Information for a Climate Smart. Nation.

[11] Lindsey R. (2022). Climate change: global sea level, NOAA Climate.gov, Science and Information for a Climate Smart. Nation.

[12] Alam M., Yasin S.K.M., Gain M., Mondal S. (2014). Renewable energy sources (res): an overview with Indian context. ISSN:2319- 7242. Int J Eng Comput Sci.

[13] Hartmann H., Ahring B.K. (2005). Anaerobic digestion of the organic fraction of municipal solid waste: influence of co-digestion with manure. Water Res..

[14] Rasheed R., Yasar A., Wang Y., Tabinda A.B., Ahmad S.R., Tahir F., Su Y. (2019). Environmental impact and economic sustainability analysis of a novel anaerobic digestion waste-to-energy pilot plant in Pakistan. Environ. Sci. Pollut. Control Ser..

[15] Gaete-Morales C., Gallego-Schmid A., Stamford L., Azapagic A. (2019). Life cycle environmental impacts of electricity from fossil fuels in Chile over a ten-year period. J. Clean. Prod..

[16] Adu I.K., Sugiyama H., Fischer U., Hungerbühler K. (2008). Comparison of methods for assessing environmental, health and safety (EHS) hazards in early phases of chemical process design. Process Saf. Environ. Protect..

[17] Diwekar U., Shastri Y. (2011). Design for environment: a state-of-the-art review. Clean Technol. Environ. Policy.

[18] Shinde A.M., Dikshit A.K., Odlare M., Thorin E., Schwede S. (2021). Life cycle assessment of bio-methane and biogas-based electricity production from organic waste for utilization as a vehicle fuel. Clean Technol. Environ. Policy.

[19] Berglund M., Börjesson P. (2006). Assessment of energy performance in the life-cycle of biogas production. Biomass Bioenergy.

[20] Ishikawa S., Hoshiba S., Hinata T., Hishinuma T., Morita S. (2006). Evaluation of a biogas plant from life cycle assessment (LCA). Int. Congr..

[21] Bühle L., Stülpnagel R., Wachendorf M. (2011). Comparative life cycle assessment of the integrated generation of solid fuel and biogas from biomass (IFBB) and whole crop digestion (WCD) in Germany. Biomass Bioenergy.

[22] Alessandra F., Jacopo B., Marco F., Adisa A. (2016). Life cycle environmental impacts of electricity from biogas produced by anaerobic digestion. Front. Bioeng. Biotechnol..

[23] Hijazi O., Munro S., Zerhusen B., Effenberger M. (2016). Review of life cycle assessment for biogas production in Europe. Renew. Sustain. Energy Rev..

[24] Esteves E.M.M., Herrera A.M.N., Esteves V.P.P., Morgado C.R.V. (2019). Life cycle assessment of manure biogas production: a review. J. Clean. Prod..

[25] Thyø K.A., Henrik W. (2007).

[26] Afrane G., Ntiamoah A. (2011). Comparative life cycle assessment of charcoal, biogas, and liquefied petroleum gas as cooking fuels in Ghana. J. Ind. Ecol..

[27] Hahn H., Hartmann K., Bühle L., Wachendorf M. (2015). Comparative life cycle assessment of biogas plant configurations for a demand oriented biogas supply for flexible power generation. Bioresour. Technol..

[28] ISO 14040:2006 (EN) (2006). Environmental management - life cycle assessment - principles and framework. https://www.iso.org/standard/37456.html.

[29] ISO 14044:2006 (EN) (2006). Environmental management- life cycle assessment- requirements and guidelines. http://www.iso.org/iso/catalogue_detail?csnumber=38498.

[30] Poeschl M., Shane W., Philip O. (2012). Environmental impacts of biogas deployment-Part I: life cycle inventory for evaluation of production process emissions to air. J. Clean. Prod..

[31] Singh A.D., Upadhyay A., Shrivastava S., Vivekanand V. (2020). Life-cycle assessment of sewage sludge-based large-scale biogas plant. Bioresour. Technol..

[32] Mezzullo W.G., McManus M.C., Hammond G.P. (2013). Life cycle assessment of a small-scale anaerobic digestion plant from cattle waste. Appl. Energy.

[33] Rebitzer G., Ekvall T., Frischknecht R., Hunkeler D., Norris G., Rydberg T., Schmidt W.P., Suh S., Weidema B.P., Pennington D.W. (2004). Life cycle assessment: Part 1: framework, goal and scope definition, inventory analysis, and applications. Environ. Int..

[34] Guinee J.B., Gorree M., Heijungs R., Huppes G., Kleijn R., Koning A. (2001).

[35] Kumawat R., Gidwani L., Rana K.B. (2022). Proc. 3rd International Conference on Recent Advances in Bio-Energy Research – 2022.

[36] Huijbregts M.A.J., Thissen U., Guinee J.B., Jager T., Kalf D., van de Meent D., Ragas A.M.J., Sleeswijk A.W., Reijnders L. (2000). Priority assessment of toxic substances in life cycle assessment. Part I: calculation of toxicity potentials for 181 substances with the nested multi-media fate, exposure and effects model USES-LCA. Chemosphere.

[37] Stewart G.J., Nelson B.S., Acton W.J.F., Vaughan A.R., Hopkins J.R., Yunus S.S.M., Hewitt N., Wild O., Nemitz E., Gadi R., Sahu L.K., Mandal T.K., Gurjarj B.R., Rickard A.R., Lee J.D., Hamilton J.F. (2021). Emission estimates and inventories of non-methane volatile organic compounds from anthropogenic burning sources in India. Atmos. Environ. X.

[38] Chen S., Chen B., Song D. (2012). Life-cycle energy production and emissions mitigation by comprehensive biogas-digestate utilization. Bioresour. Technol..

[39] Tambone F., Scaglia B., D'Imporzano G., Schievano A., Orzi V., Salati S., Adani F. (2010). Assessing amendment and fertilizing properties of digestates from anaerobic digestion through a comparative study with digested sludge and compost. Chemosphere.

[40] Yasar A., Rasheed R., Tabinda A.B., Tahir A., Sarwar F. (2017). Life cycle assessment of a medium commercial scale biogas plant and nutritional assessment of effluent slurry. Renew. Sustain. Energy Rev..

[41] Tao M., Cheng W., Nie K., Zhang X., Cao W. (2022). Life cycle assessment of underground coal mining in China. Sci. Total Environ..

[42] Yin L., Liao Y., Zhou L., Wang Z., Ma X. (2017). Life cycle assessment of coal-fired power plants and sensitivity analysis of CO_2_ emissions from power generation side. IOP Conf. Ser. Mater. Sci. Eng..

[43] Rasheed R., Javed H., Rizwan A., Sharif F., Yasar A., Tabinda A.B., Ahmad S.R., Wang Y., Su Y. (2021). Life cycle assessment of a cleaner supercritical coal-fired power plant. J. Clean. Prod..

[44] Ingrao C., Rana R., Tricase C., Lombardi M. (2015). Application of carbon footprint to an agro-biogas supply chain in southern Italy. Appl. Energy.

[45] Ingrao C., Bacenetti J., Adamczyk J., Ferrante V., Messineo A., Huisingh D. (2019). Investigating energy and environmental issues of agro-biogas derived energy systems: a comprehensive review of Life Cycle Assessments. Renew. Energy.

[46] Yadav S., Prakash R. (2014). Status and environmental impact of emissions from thermal power plants in India. Environ. Forensics.

